# A superior bright NIR luminescent nanoparticle preparation and indicating calcium signaling detection in cells and small animals

**DOI:** 10.1186/s13578-018-0235-1

**Published:** 2018-06-05

**Authors:** Jian Zhang, Joseph. R. Lakowicz

**Affiliations:** 1 Department of Biochemistry and Molecular Biology, Center for Fluorescence Spectroscopy, University of Maryland School of Medicine, 725 West Lombard Street, Baltimore, MD 21201 USA; 2 Present Address: Vigene Biosciences Inc., 9430 Key W. Ave Suite 105, Rockville, MD 20850 USA

**Keywords:** Gold nanorod (AUNR), Indocyanine green (ICG), Dual-mode plasmons, Near-field fluorescence (NFF), Luminescent nanoparticle (LNP), Fluorescence imaging, Imaging contrast agent

## Abstract

**Background:**

Near-field fluorescence (NFF) effects were employed to develop a novel near-infrared (NIR) luminescent nanoparticle (LNP) with superior brightness. The LNP is used as imaging contrast agent for cellular and small animal imaging and furthermore suggested to use for detecting voltage-sensitive calcium in living cells and animals with high sensitivity.

**Results:**

NIR Indocyanine green (ICG) dye was conjugated with human serum albumin (HSA) followed by covalently binding to gold nanorod (AuNR). The AuNR displayed dual plasmons from transverse and longitudinal axis, and the longitudinal plasmon was localized at the NIR region which could efficiently couple with the excitation and emission of ICG dye leading to a largely enhanced NFF. The enhancement factor was measured to be about 16-fold using both ensemble and single nanoparticle spectral methods. As an imaging contrast agent, the ICG–HSA-Au complex (abbreviate as ICG-Au) was conjugated on HeLa cells and fluorescence cell images were recorded on a time-resolved confocal microscope. The emission signals of ICG-Au complexes were distinctly resolved as the individual spots that were observed over the cellular backgrounds due to their strong brightness as well as shortened lifetime. The LNPs were also tested to have a low cytotoxicity. The ICG-Au complexes were injected below the skin surface of mouse showing emission spots 5-fold brighter than those from the same amount of free ICG–HSA conjugates.

**Conclusions:**

Based on the observations in this research, the excitation and emission of NIR ICG dyes were found to be able to sufficiently couple with the longitudinal plasmon of AuNRs leading to a largely enhanced NFF. Using the LNP with super-brightness as a contrast agent, the ICG-Au complex could be resolved from the background in the cell and small animal imaging. The novel NIR LNP has also a great potential for detection of voltage-gated calcium concentration in the cell and living animal with a high sensitivity.

## Background

Calcium is a well-known signaling ion in most eukaryotes [1, 2]. A calcium concentration gradient across a plasma membrane and intracellular organelle can fluxes dynamically via orchestrated channel openings, and furthermore generate tightly controlled spatial and temporal patterns. In electrically excitable neurons and muscle cells, voltage-gated calcium channels are coupled with the membrane depolarization due to the calcium influx, which can significantly alter the cellular physiology [3, 4]. Hence, it is of importance to understand the calcium concentration gradient and fluctuation in the cells. This study might also highlight the crucial role of calcium single at a cellular level as well as in living animals.

Currently, the voltage-gated calcium channels in the cells, tissues and mediums are often measured by a fluorescence imaging [5, 6]. Typically, a fluorophore is used as a calcium indicator to chelate with a calcium ion creating a fluorescence signal. With a change of concentration or environment of Ca^2+^ ions in the cell, the fluorescence signal from the indicator is altered. This method can be also used for exploring the intracellular calcium concentration and gradient of calcium ion at the cellular level as well as in the living animals [7, 8]. Actually, the monitoring voltage-gated imaging calcium has become an important topic in the calcium channel detection because the calcium signals exert their highly specific functions in the well-defined cells or/and small animals.

In the past decades, novel calcium indicators have been synthesized as the organic compounds [9, 10]. Most of these calcium indicators have their emission wavelengths at visible region. It is known that the fluorescence signals at the visible region have severe interference from the strong backgrounds from cellular autofluorescence and light scattering in the biological systems [11–17]. To suppress the interference, a near-infrared (NIR) fluorophore is suggested for using as imaging contrast agents [18–20]. Tissue and water have a window with a low background allowing a penetration of excitation light deeper into the tissue and allow the detection of emissions from the fluorophores with a better resolution with the cells and tissues.

However, as imaging contrast agents, the NIR fluorophores have their two significant drawbacks: (1) low absorption coefficients which may result in their low brightness and (2) low photostability that results in their short bleaching time [21, 22]. To our knowledge, there is still lack of an efficient contrast agent that allows the detection of calcium ions in the cells and small animals at the single molecule level. Hence, there is an essential need for a new approach that can greatly improve the fluorescence properties of NIR fluorophores particularly on their brightness and photostability.

Near-field fluorescence (NFF) can improve the fluorescence properties of fluorophores [23]. Fundamentally, a metal nanoparticle can create a local electromagnetic field nearby as a light irradiation and the electromagnetic field is confined into the metal plasmons [24–26]. When a fluorophore is localized within the near-field range from the metal nanoparticle surface, the excitation/emission of fluorophore can strongly couple with the light-induced plasmons on the metal nanoparticles [27, 28], and the excitation or/and emission rates of fluorophore can be significantly increased. As a result, the fluorescence properties of fluorophore can be greatly improved including (1) largely enhanced emission intensity and quantum yield (2) extended photobleaching time and (3) reduced photoblinking of fluorophore [28].

Since the spherical metal nanoparticle with a reasonable size displays its single-mode plasmons at the visible range most of current NFF effects were tested using the visible fluorophores, and only few using NIR fluorophores [29–33]. Different from the spherical metal nanoparticles, the shaped metal nanoparticles, such as metal nanoshells or nanorods, can display their surface plasmons at longer wavelength [34, 35]. For instance, the gold nanorods (AuNRs) can display their split dual plasmons from the short (transverse) and long axis (longitudinal), respectively [36–38], and importantly, the longitudinal plasmon can be tuned to the NIR region by adjusting the aspect ratio of AuNRs. Thus, the longitudinal plasmons from the AuNRs are expected to be able to sufficiently couple with the excitation/emission of NIR fluorophores leading to a strong NFF-induced fluorescence at the NIR region. Meanwhile, the NIR AuNRs remain have reasonable sizes.

We are interested in developing novel NIR LNPs with high brightness and furthermore using these LNPs as imaging contrast agents, for determining the calcium ions in the cells and living small animals. In this study, the NFF effect was employed to prepare the novel NIR LNPs. Indocyanine green (ICG) is a FDA-proved nontoxic NIR fluorophore for patient safety in ophthalmology [39, 40], and also known as a voltage-sensitive fluorophore that can be used to determine the voltage-gated calcium channels by adding chelators on its chemical structure [41]. In this study, the ICG dye was bound to AuNRs within a near-field distance to explore the NFF at the NIR region.

Briefly, the ICG dyes were first conjugated in human serum albumin (HSA) followed by covalently binding the conjugates on the surfaces of AuNRs [42, 43]. Since the HSA molecules have an average size of *ca.* 10 nm, the ICG dyes conjugated to the HSA molecules are distributed within a near-field distance from the surfaces of AuNRs. In addition, the excitation/emission of ICG dyes can sufficiently couple with the longitudinal plasmons of AuNRs, and thus, a NFF from the bound ICG dyes on the AuNRs was expected to occur. The ensemble and single nanoparticle spectra were used to evaluate the change of optical properties of ICG dyes prior to and after their binding on the AuNRs. Using as a nanoparticle contrast agent, the ICG–HSA-Au (abbreviated as ICG-Au) complex was bound to HeLa cells and the fluorescence cell images were collected for evaluating the fluorescence spectral properties at the single nanoparticle level. The ICG-Au complex was also injected into the mouse for live animal fluorescence tomography. Comparing with the free ICG–HSA conjugates, the ICG-Au complex displayed significantly improved properties for the live animal tomography uses [44–50].

## Results

### ICG–HSA conjugates

In this research, the NIR luminescent nanoparticle was developed using a strong NFF effect by binding the NIR ICG dyes on the surfaces of AuNRs within a near-field distance. Thus, the ICG dyes were first conjugated with the HSA molecules to form the ICG–HSA conjugates, and the conjugates were then covalently bound onto the surfaces of AuNRs. In the experiments, the ICG and HSA were codissolved in an aqueous solution with a molar ratio of ICG/HSA = 4/1. After the reaction, the free ICG dyes were removed from solution by a dialysis against water.

**Fig. 1:**
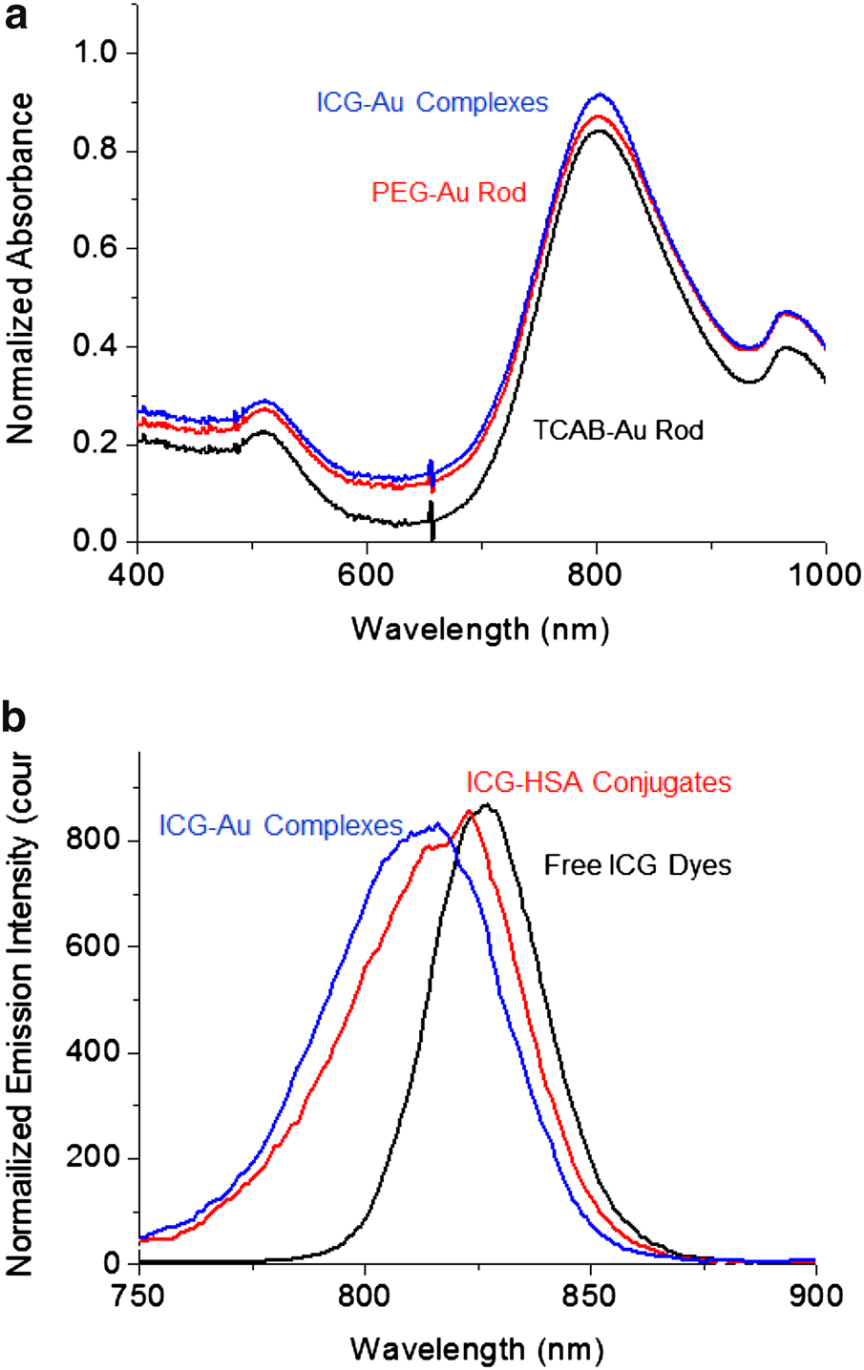
**a** Absorption spectra of AuNRs as CTAB-coated, PEG-coated, and ICG–HSA conjugate-bound in a 10 mM PBS buffer solution. **b** Ensemble emission spectra from the ICG dyes as free, conjugates in HSA, and complexes with AuNRs in a 10 mM PBS buffer solution

The fluorescence properties of ICG dyes before and after the conjugation were measured using ensemble spectroscopy. Upon excitation at 760 nm, the ICG–HSA conjugate was observed to exhibit an emission band centered at 819, 7 nm shifting to shorter in comparison with the free ICG dyes in aqueous solution (Fig. 1a). The emission band also became broader with the ICG conjugation, which may be due to the plasmons or the short emission wavelength of ICG.

### Covalently binding ICG–HSA conjugates on AuNRs

The biological properties of nanoparticles, such as cell uptake and circulation time, are known to strongly rely on their surface properties [51, 52]. In this study, AuNRs were prepared with the protection of cetyltrimethylammonium bromide (CTAB) monolayers on the surfaces. To improve their bioactivity, the CTAB-monolayers on the AuNRs were replaced with the thiolate polyethylene glycol (PEG) monolayers via a surface substitution reaction on the nanoparticle. The free small molecules were removed by a dialysis against water. Most CTAB molecules on the AuNR surfaces were supposed to replace by the PEG molecules. The change of monolayers on the AuNR surfaces could be reflected by the solubility change of AuNRs in aqueous solution prior to and after the reaction. In addition, since these PEG molecules were bound on the AuNR surfaces via sulfur-metal bonds, much stronger than the CTAB molecules via electrostatic interactions, the PEG-AuNRs should become more chemically stable in solution [51, 52].

To bind the ICG–HSA conjugates on the AuNRs, the PEG monolayers on the AuNRs were partially substituted by thiolate carboxyl-ligand of *N*-(2-mercapto-propinyl)glycine ligands to create the reactive sites on the AuNR surfaces via surface exchange reaction [53, 54]. Experimentally, the thiolate carboxyl-ligand was dissolved in solution with a molar ratio of carboxyl ligand/AuNR = 100/1. After the substitution reaction, the unsubstituted ligands were removed by a dialysis against water.

The ICG–HSA conjugates were covalently bound on the AuNRs via the surface condensation of primary amino moieties in the ICG–HSA conjugates with the carboxyl moieties on AuNRs in the presence of 1-(3-dimethylaminopropyl)-3-ethylcarbodiimide hydrochloride (EDC) as the condensation agent. The ICG–HSA conjugates were dissolved in excess amount in solution to avoid aggregation of nanoparticles through the crosslinking. The final AuNR product was recovered by a centrifugation and then purified by a dialysis against water.

### Evaluation of ICG-Au complex by microscope and ensemble spectroscopy

**Fig. 2:**
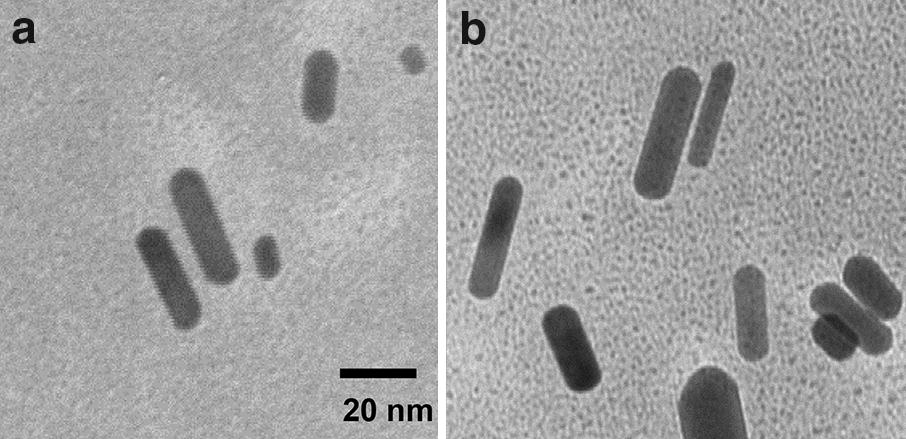
TEM images of (**a**) CTAB-AuNRs and (**b**) ICG-Au complexes

Tomography of AuNRs through the surface reactions was evaluated using a transmission electron microscope (TEM). Representative images of AuNRs are shown in Fig. 2a, b prior to and after the surface reactions on the AuNRs. These AuNRs were observed to have an average width of 10 nm and an average length of 40 nm and the aspect ratio was calculated to be *ca.* 4.0. There was no significantly change on the tomography with the three-step surface reactions on AuNRs, reflecting that the surface reactions on the AuNRs only altered the monolayer composition on their surfaces but not on their metal cores.

The plasmon absorption of metal nanoparticle is known to be sensitive to the composition of monolayers on the surface [30, 31]. In this study, the absorption spectrum was used to measure the replacement of ligands on the AuNR surfaces. The AuNRs displayed a dual plasmons from the short (transverse) and long axis (longitudinal) at 504 and 802 nm, respectively (Fig. 1a). Following the monolayer reactions on the surfaces of nanoparticles, the two plasmons bands were found to remain, but the maxima were slightly shifted to longer at 511 and 807 nm (Fig. 1a), respectively.

Ensemble fluorescence spectra were also sensitive to the binding of ICG dyes on the metal nanoparticle surfaces. It was shown that the emission band of ICG-Au complexes was centered at 814 nm (Fig. 1b), 5 nm shifting to shorter in comparison with the free ICG–HSA conjugates. Fluorescence spectral shifts have been attractive to the wavelength dependent on the interaction of metal nanoparticle and fluorophore [55–57].

### Evaluation of ICG-Au complex by single nanoparticle spectroscopy

**Fig. 3:**
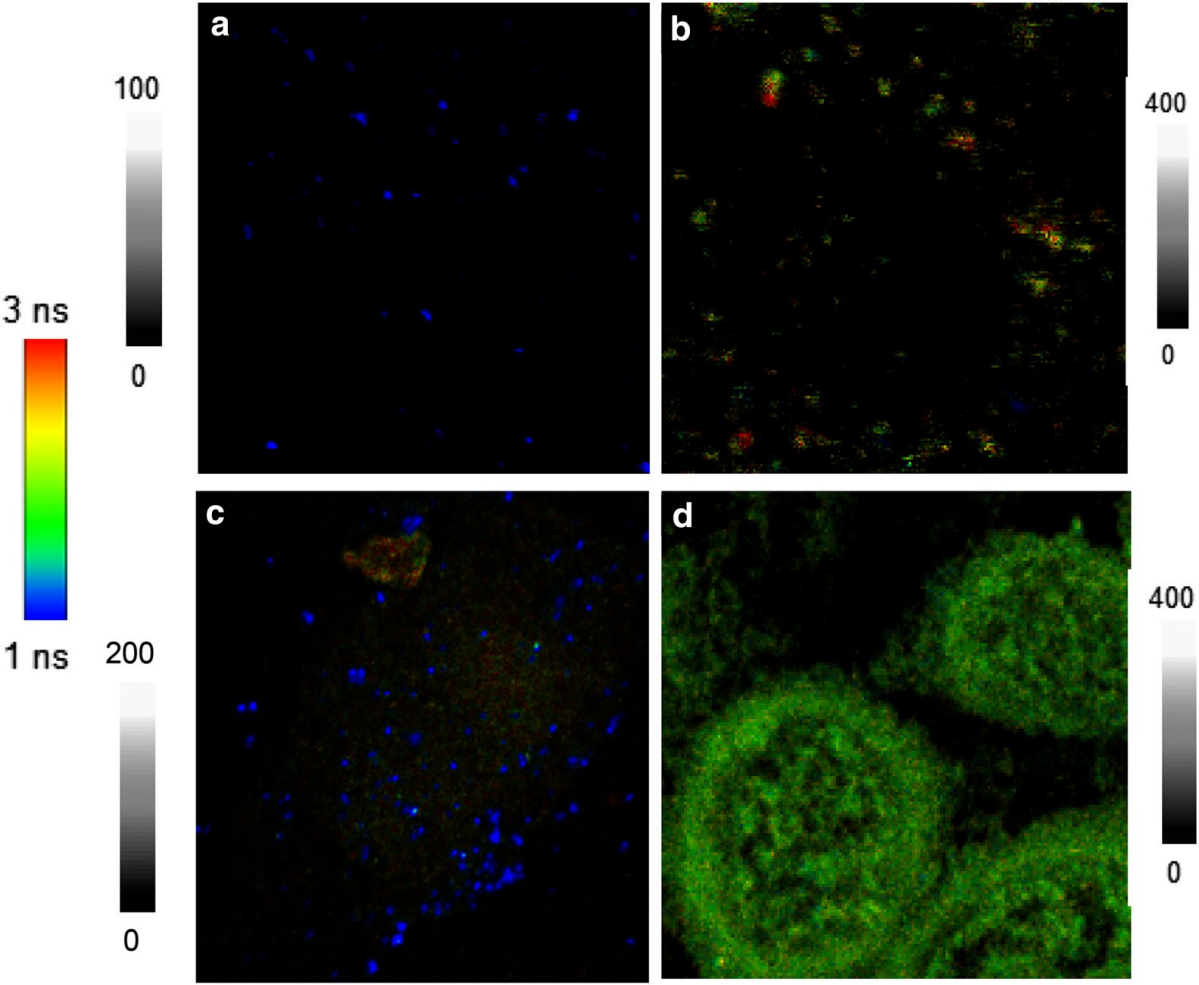
Upper panels represent the emission imaging from (**a**) ICG-Au complexes and (**b**) ICG–HSA conjugates. Diagrams are 5 × 5 µm and resolutions are 100 × 100 pixels with an integration of 0.6 ms/pixel. Bottom panels represent fluorescence images from the cells conjugated with (**c**) ICG-Au complexes and (**d**) ICG–HSA conjugates. Diagrams are 50 × 50 µm and resolutions are 100 × 100 pixel with an integration of 0.6 ms/pixel. The samples were excited with a 640 nm laser. Note the different intensity scales. The images of **a** and **c** were collected with a laser power 10-fold less than the images of **b** and **d**

**Fig. 4:**
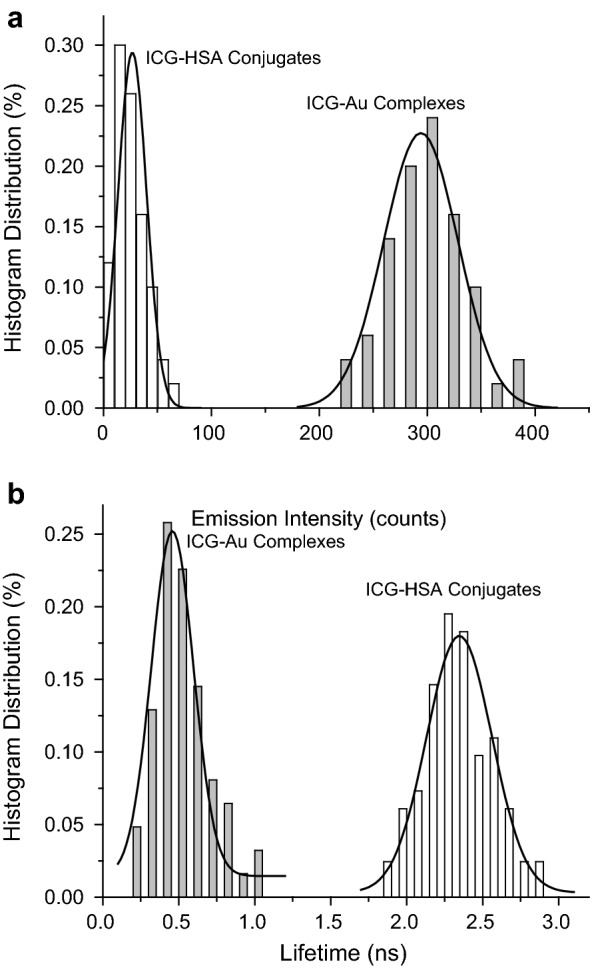
Histogram distributions of single sots of (**a**) emission intensities and (**b**) lifetimes from the ICG–HSA conjugates as free and bound on the AuNRs

In addition to the ensemble spectrum, NFF effect on the ICG-Au complexes could be evaluated using single nanoparticle spectral measurement. To prepare the test samples, the ICG-Au complex was diluted to nM in aqueous solution and then cast a drop on a glass coverslip followed by drying in air. With a low concentration in solution before drying, the ICG-Au complexes were mostly existed as isolated particles on the coverslip. The single nanoparticle measurements were performed on a time-resolved confocal microscope. Upon excitation with a 640 nm laser, both the emission intensities and lifetimes from the ICG-Au complexes (as shown in Fig. 3a) were collected at single nanoparticle level [54]. As control, the free ICG–HSA conjugates were also diluted in solution and cast on the coverslip. The emission signals were collected with the same conditions on the confocal microscope but with an excitation power of laser 10-fold stronger. The collected emission spots from the free conjugates were much dim as shown in Fig. 3b demonstrating lower emission intensities of free conjugates. For each sample, at least 50 emission spots were collected, and the histogram of the intensities and lifetimes were obtained by fitting with a Gaussian distribution curve (Fig. 4a for the intensity and b for the lifetime), and the maximum values of curves were obtained to represent the emission intensity and lifetime of sample, respectively.

The near-field interaction of an excited fluorophore with a metal nanoparticle may increase the radiative rate of fluorophore, and as a result, the lifetime of fluorophore can be decreased [25]. Hence, the lifetime can be used as an important parameter to evaluate the near-field interaction. Herein, the decays of excited ICG-Au complexes as well as ICG–HSA conjugates were recorded using the confocal microscope following by fitting with a Gaussian distribution curve (Fig. 4b). The maximum values of lifetime of ICG dyes were obtained, showing a significant decrease of lifetime from 2.3 ns for the unbound ICG–HSA conjugates to 0.4 ns for the ICG-Au complexes.

### Fluorescence cellular imaging

To test the fluorescence properties of ICG-Au complexes for the cell imaging, the ICG-Au complexes were used as an imaging contrast agent to conjugate with HeLa cells. Briefly, HeLa cells were cultured on coverslips followed by fixing using 4% paraformaldehyde. The cell-fixed coverslip were incubated with the ICG-Au complex for 30 min and then completely washed with PBS buffer. Fluorescence cell images were collected on the time-resolved confocal microscope in both the intensity and lifetime. A representative image was presented in Fig. 3c. It was shown that the ICG-Au complexes were presented as individual spots on the cells distinctly observable from the cellular backgrounds either due to their strong intensity and differentiated lifetime.

As control, the ICG–HSA conjugates were also conjugated with HeLa cells, and the cell images were recorded on the confocal microscopy with the same conditions (Fig. 3d). Comparing with the images of blank cells, the overall cell images became brighter, indicating that the ICG–HSA conjugates were indeed conjugated on the cells. But the emission signals from the single ICG–HSA conjugates could not be well resolved as individual spots from the cellular backgrounds of cell images, which was due to their low brightness as well as a lifetime close to the cellular background.

### Cytotoxicity measurements

**Fig. 5:**
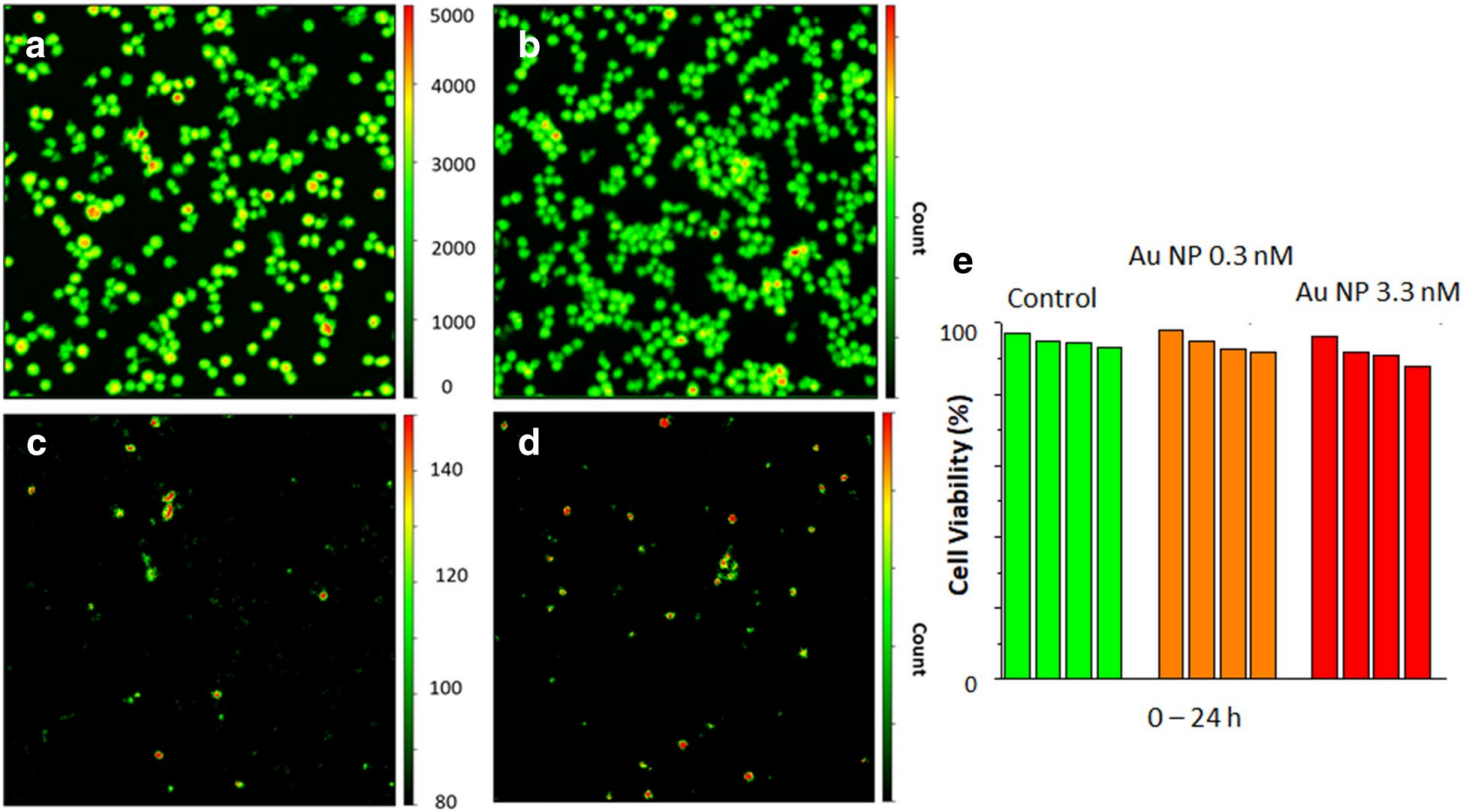
The panel of cell images of live HeLa cells stained with calcein AM without (**a**) the Au nanoparticle and (**b**) in the presence of 3 nM of Au nanoparticles. The images of calcein stained cells (**a**) and (**b**) were acquired after 24 h of nanoparticle treatments upon excitation with a 443 laser diode and at bandpass filter 514/30 nm. The images of **c** and **d** represent the autofluorescence of cells without (**a**) the Au nanoparticle and (**b**) in the presence of 3 nM of Au nanoparticles after 24 h. The autofluorescence images of cells were collected upon an excitation at 640 nm and with a longpass filter of 655 nm. Cells with brighter autofluorescence in **c** and **d** are classified as dead. **e** represents rates of viable cells in the presence of 0.3 and 3 nM in the cell medium as well as in the absence of Au nanoparticle as the control at time interval = 0.5, 2, 12, 24 h

**Fig. 6:**
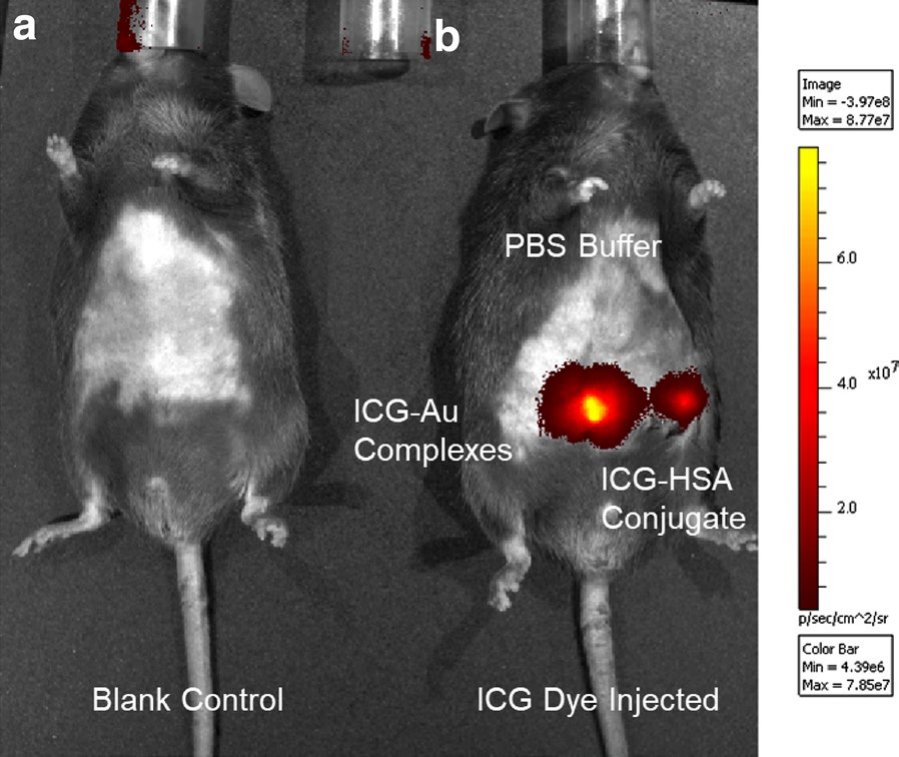
In-vivo fluorescence tomography images of mice using the ICG-Au complexes as contrast agent injected below the skin of mice. The images were collected on a Xenogen IVIS-200 small animal tomography system with a bandpass filter from 665 to 695 nm for the background, a filter from 710 to 760 on the excitation side, and a filter from 810 to 875 nm on the emission side. A 750 nm laser was used as the excitation source. Total photon flux (photons/s) was calculated and corrected for tissue depth by spectral imaging using Living Image 3.0 software (Xenogen). The left image (**a**) was collected on a control mice and the right image (**b**) was collected by injection with the ICG-Au complex, ICG–HSA conjugate, or a blank PBS buffer solution with the same volume on the same mice

Cytotoxicity of free conjugate and ICG-Au complex were tested on live HeLa cell using calcein AM assay. The cell images at different time intervals were collected on the time-resolved confocal microscope as shown in Fig. 5. An area with a large number of cells was selected for statistical analysis for the cell survival. The live cells could be identified as stained with calcein AM (green cell viability stain) as shown in the image A when there was in the absence of nanoparticle (294 cells) and in the image B when there was in the presence of 3 nM Au nanoparticles (207 cells) after the treatment time of 24 h. The number of cell with high autofluorescence was counted as 20 in the absence of nanoparticles (Fig. 5c) and as 13 in the presence of Au nanoparticles (Fig. 5d), showing that the rates of viable cells are 93.1 and 93.7%, respectively. The results in the presence of 0.3 and 3 nM as well as the control were listed in Fig. 6e reflecting that the presence of Au nanoparticles in the cell medium had only a slight influence to the cells survival. It also demonstrates that the Au nanoparticles have very low cytotoxicity.

### Fluorescence small animal imaging

Six 5–6 weeks nude mice were selected to test the optical properties of novel LNP by small animal fluorescence imaging. The mice were randomly divided into two groups and each group had three mice. The mice were first euthanized under deep anesthesia following by injecting the ICG-Au complex below the skin surface of mouse [16, 17]. As control, the ICG–HSA conjugate solution containing the same amount of ICG dye was also injected in the same mouse at a different site. Fluorescence small animal imaging was performed on a Xenogen IVIS-200 system and the representative images were shown in Fig. 6. An image from an untreated mouse was also presented as control. The emission spots from the injection sites by the ICG-Au complex and ICG–HSA conjugate were observed to be significantly different: the spot by the ICG-Au complex was *ca*. 5-fold brighter than the spot by the ICG–HSA conjugate. Since the two injection sites on the same mice were known to contain the same amounts of ICG dye, the difference on their brightness should be due to their different emission intensities. In the other words, an enhanced fluorescence of NFF from the ICG-Au complex results in increased brightness of ICG dyes in the small animal imaging.

## Discussion

In this study, a NIR LNP was designed and prepared on the basis of NFF effect. The ICG dyes were conjugated with the HSA molecules followed by covalently binding on the AuNRs [42, 43]. To achieve the ICG–HSA conjugates with the maximal brightness, the molar ratio of ICG over HSA in the conjugation was controlled to be 4/1 in the reaction. Too many ICG dyes on one HSA molecule would result in self-quenching among the fluorophores and too few dyes in one HSA molecule would result in a low brightness.

To improve the bioactivity of nanoparticles, the CTAB monolayers on the AuNRs were replaced by the PEG monolayers via surface substitution reaction [51, 52]. Most of the CTAB molecules on the AuNRs were believed to replace by the PEG molecules, and supported by a change on the solubility of AuNRs in aqueous solution prior to and after the replacement. Prior to the replacement, the AuNRs were found to have a very good dispersion in water, whereas after the replacement, the AuNRs were readily stuck on the wall of glass tube, which was due to increased hydrophobicity of nanoparticle surfaces by the PEG monolayers. In addition, with stronger sulfur-metal covalent bonds of PEG with the AuNRs, the modified AuNRs were supposed to have an improved chemical stability in solution [51, 52].

**Fig. 7:**
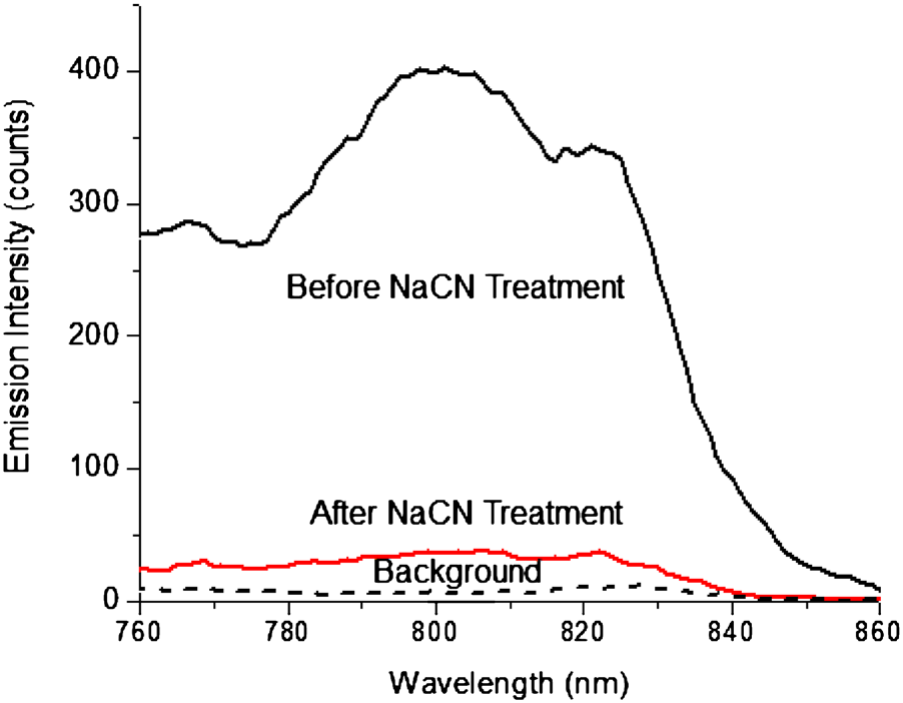
Emission spectral change of ICG-Au complex in 10 mM PBS buffer solution before and after a NaCN treatment

To covalently bind the ICG–HSA conjugates on the AuNRs, the PEG monolayers on the AuNRs were partially substituted by *N*-(2-mercapto-propinyl)glycine to create reactive sites on the nanoparticle surfaces. The ICG–HSA conjugates were then covalently bound on the AuNRs via a condensation reaction [53]. The binding of ICG–HSA conjugates on the AuNRs could be supported by a change of absorption and fluorescence spectra prior to and after reactions as described early. The binding number of ICG–HSA on each AuNR could be measured using a NaCN treatment method [53]. Typically, several drops of 0.1 N NaCN aqueous solution were added into 0.5 nM ICG-Au complex solution. It was observed that the plasmon color of solution disappeared progressively with the time, showing that the metal nanoparticles were dissolved by NaCN. As a result, the ICG–HSA conjugates were released from the nanoparticles as free into the solution. The whole process could be monitored by the ensemble fluorescence spectrum expressing a dramatic decrease of emission intensity (Fig. 7) until saturation. The ICG–HSA conjugates were released as free in the solution completely lost the NFF effect leading to a dramatic decrease on the emission intensity [31]. Using the saturated emission intensity, the concentration of ICG–HSA conjugates in the solution was measured to be 3 × 10^−9^ M. Since the amount of ICG–HSA was not significantly changed in solution prior to and after the NaCN treatment, according to a ratio of emission intensity prior to the treatment over that after the treatment, the enhancement factor for the ICG dye on AuNR was calculated to be 16.3.

To evaluate the NFF effect of NIR dyes on the AuNRs, 20 and 50 nm gold nanospheres were prepared following by covalently binding with the ICG–HSA conjugates via the same strategy. Using the NaCN treatment, the enhancement factor of ICG dyes on the 50 nm gold nanospheres was measured to be 2.3, much lower than that on the AuNRs, although a 50 nm gold nanosphere is almost 20-fold larger on the volume than a AuNR. The ICG dyes on a 20 nm gold nanosphere, which has an approximately identical volume to a AuNR, resulted in an insignificant NFF effect. The nanospheres did not display a plasmon band at the NIR region, and as a result, could not sufficiently couple with the excitation and emission of ICG dyes. In contrast, the AuNRs displayed a NIR longitudinal plasmon band leading to their sufficiently coupling with the excitation and emission of ICG dyes, and thus, resulted in a strong NFF effect. This result indicates that the longitudinal plasmon band from a shaped metal nanoparticle is very important for its sufficient coupling with a NIR fluorophore and brings up a strong NFF in the NIR region.

The ICG-Au complexes were also evaluated at single nanoparticle level on a time-resolved confocal microscope. The ICG-Au complexes were found to have an intensity 10.5-fold higher than the free ICG–HSA conjugates. Since the emission of ICG-Au complexes were collected upon excitation with a laser power of 10-fold lower than those of ICG–HSA conjugates, the ICG-Au complexes were calculated to be 105-fold brighter than the ICG–HSA conjugates. Considering that one AuNR was averagely bound with 6 ICG–HSA conjugates, the enhancement factor per ICG molecule was estimated to be 16.7, very close to the enhancement factor achieved on the ensemble spectra. This enhancement factor is also comparable with the value from some visible fluorophores on the metal nanospheres [41], representing a sufficient NFF interaction of NIR fluorophores with the AuNRs.

Besides the emission intensity, the near-field effect of a fluorophore with a metal nanoparticle may result in a largely reduced lifetime [25]. In this study, the lifetimes were collected at single nanoparticle level on a confocal microscope, and the histogram of lifetimes was fitted with a Gaussian distribution. A maximum was obtained at 0.4 ns (Fig. 4b), much shorter than the lifetime of unbound ICG–HSA conjugates at 2.3 ns, supporting an efficient near-field coupling of ICG molecules with the AuNRs. It was interesting to notice that the lifetime of ICG-Au complex was beyond the range of autofluorescence (2–5 ns) in the lifetime fluorescence cell images, which would beneficiate to isolate the emission signals of novel NIR nanoparticle fluorophores from the cellular backgrounds on the time-resolved images [44, 45].

Using as an imaging contrast agent, the ICG-Au complexes were conjugated with HeLa cells for fluorescence cell imaging. Fluorescence cell images were recorded on the confocal microscope with both the intensity and lifetime. It was shown that the emissions signals from the ICG-Au complexes were distinctly isolated as individual spots from the cellular backgrounds (Fig. 3c). The intensity ratio of signal/noise on the image was estimated to be approx. 63, much higher than the value from the most organic fluorophores or LNPs, which was due to high brightness of ICG-AuNRs [44, 45]. In addition, because of largely shortened lifetime, the emissions of ICG-AuNRs could be better resolved from the cellular backgrounds on the lifetime cell images.

As control, the HeLa cells were also incubated with the free ICG–HSA conjugates, and the cell images were recorded under the same conditions (Fig. 3d). It was shown that the overall cell images became brighter than the images of blank cells without the treatment supporting that the ICG–HSA conjugates had been conjugated with the cells. But the emissions from the ICG–HSA conjugates could not be resolved as individuals from the cellular backgrounds, which was due to their low brightness as well as close lifetime relative to the cellular autofluorescence at the backgrounds.

In this study, the LNPs were not functionalized with the specific bioactive molecules. Thus the ICG-Au complexes were supposed to randomly distribute through the cells (Fig. 3c). On the other hand, because of bulky sizes of ICG-Au complexes and relatively short incubation time, these LNPs were observed to mostly attach on the cell surfaces, which could be the result of short incubation time with the cells. Our other experiments (not shown herein) also demonstrated that the metal nanoparticles of this size can penetrate through the cell membrane and enter the cells. We will use these LNPs as indicators to detect the calcium channels and concentration gradients by the fluorescence cell imaging. Once the nanoparticles are functionalized with the bioactive molecules and then enter the cells, they will become possible to label the target molecules with a higher efficiency because of the presence of multiple functional groups on their surfaces.

Cytotoxicity of ICG-Au complexes was tested on live HeLa cell using a calcein AM assay showing that the luminescent nanoparticles have only slight or even insignificant cytotoxicity to the live HeLa cell. It is known that the cytotoxicity of metal nanoparticles strongly relies on the coating layers on the metal cores. For a relatively low cytotoxicity of metal nanoparticles in this study, it can be described by two possible factors. First, polyethylene glycol layers were coated on the Au cores via covalent bonds. These covalent bonds are much stronger than the statistic interactions that the nanoparticles are generally bound by leading to the current ICG-Au complexes are more chemically stable in the cell medium or animal bodies. Second, the ICG dyes have low toxicity. Hence, the ICG-Au complexes can affect insignificantly or slightly the cells viability as observed in this study.

To test the imaging function, the ICG-Au complex was injected to the skin surface of mice for the fluorescence small animal imaging [16, 17]. It was shown that the emission spot from the injection site by the ICG-Au complex was *ca*. 5-fold brighter than the site by the ICG–HSA conjugate. Since the two injection sites contained the same amounts of ICG dye, the difference of the brightness of two spots over the mice image should be due to the different brightness between the ICG-Au complex and free ICG–HSA conjugate.

However, it was noticed that 5-fold increased fluorescence intensity of LNP over the free ICG–HSA conjugate on the mouse was smaller than the enhancement factor of 16-fold for the ICG dyes on the AuNRs. This value was also much less than the difference of brightness for the LNP over the free ICG–HSA conjugates in the fluorescence cellular imaging. It was probably due to a much stronger interference of autofluorescence background in the small animal imaging.

We are interested in developing novel NIR LNP and using it for determining the target molecules in both the cell and small animal. The immunohistochemistry of ICG-Au complex in the organs of mouse were not performed in this study, and thus, the information on the toxicity of ICG-Au complex to the small animals is not available in this paper. But it is also noticed that the mice kept good health after 1 week of ICG-Au complex injection, indicating that the ICG-Au complexes have a relatively low toxicity to these mouse [58, 59]. More research on this aspect will be conducted in our laboratory.

In this study, a superior bright NIR LNP was developed for determining the cell membrane specific targets in the cells and small animals. We are interested in the voltage-gated calcium channels in the cell, tissue, and medium as well as in the small animals. The ICG-Au complex will be used as the fluorescence indicator to explore the change of calcium ion in the cells and furthermore the intracellular calcium concentrations or gradients at the cellular level as well as in the small animals. It is important to use this LNP for single molecule detection at the cell level. But due to the strong backgrounds, the emission signals of single nanoparticles become very difficult to resolve on the small animal imaging. However, with largely enhanced fluorescence and shortened lifetime, the ICG-Au complexes can offer us a greater opportunity to insight the target calcium ions and their activities with a better resolution and a larger depth of tissue layer.

Because of instrumentation limitations, the small animal image could not be recorded in time-resolved model on the current imaging system. But we expect that with largely enhanced fluorescence and unique lifetime, the ICG-Au complex can provide us an opportunity to observe the target molecules and their activities with a better resolution and a larger depth of tissue layer in the time-resolved small animal imaging.

## Conclusions

Due to a longitudinal plasmon at the NIR region, AuNRs were demonstrated to sufficiently couple with the excitation/emission of NIR fluorophores leading to largely enhanced NFF effect. NFF could be well evaluated with both ensemble and single nanoparticle spectroscopy. Considering each AuNR was averagely bound by six ICG–HSA conjugates, a single ICG-Au complex was over 100-fold brighter than a single ICG–HSA conjugate. Strong near-field interactions could also result in shortened lifetime which is distinguished from the lifetime range of cellular autofluorescence in the fluorescence cell and small animal images. Because of its unique lifetime, the ICG-Au complex can provide us an opportunity to observe the target molecules and their activities with a better resolution and a larger depth of tissue layer in the time-resolved small animal imaging. The novel NIR nanoparticle fluorophores will be used as calcium indicators to efficiently determine voltage-sensitive fluorescence calcium signal in-vivo at single cellular level and in living small animals.

## Methods

All chemical reagents and spectroscopic grade solvents were used as received from Fisher or Sigma/Aldrich. Cardiogreen (indocyanine green, ICG) and human serum albumin (HSA) were available from Sigma/Aldrich. The gold nanorods (AuNRs) and gold nanospheres were purchased from Sigma/Aldrich. RC dialysis membrane (MWCO 4000) was obtained from Spectrum Laboratories, Inc. Nanopure water (> 18.0 MΩ cm^−1^) purified on Millipore Milli-Q gradient system was used in all experiments.

### Preparing ICG–HSA conjugates and binding conjugates on gold nanorods

Indocyanine green (ICG) was first conjugated in human serum albumin (HSA). The ICG and HSA were codissolved in 10 mM phosphate buffered saline (PBS buffer) solution at pH = 7.4. The molar ratio of ICG over HSA was 4/1 in solution. The solution was stirred at room temperature for 24 h. Free ICG dyes in solution were removed by dialysis against 10 mM PBS buffer.

The ICG–HSA conjugates were covalently bound on the gold nanorods (AuNRs). Three-step chemical reaction on the AuNR surface was employed. First, the CTAB monolayers on the AuNRs were replaced by hexa(ethyleneglycol)mono-11-(acetylthio)undecyl ether, a polyethylene glycol (PEG) ligand. 5 × 10^−11^ M commercially available AuNRs were dispersed in an aqueous solution containing 1 × 10^−5^ M hexa(ethyleneglycol)mono-11-(acetylthio)undecyl ether. The solution was continuously stirred for 12 h, and the AuNRs were recovered by centrifugation. Second, the PEG monolayers on the AuNRs were partially substituted with *N*-(2-mercapto-propinyl)glycine via surface substitution reaction. 5 × 10^−11^ M PEG-AuNRs were dispersed in an aqueous solution containing 5 × 10^−9^ M *N*-(2-mercapto-propinyl)glycine. The solution was continuously stirred for 24 h. The AuNRs were recovered by configuration. Finally, the ICG–HSA conjugates were covalently bound on the AuNRs via *N*-hydroxysuccinimide (NHS) condensation reaction. 5 × 10^−11^ M PEG-AuNRs were dispersed in 10 mM PBS buffer solution at pH 8.2 containing 5 × 10^−9^ M ICG–HSA conjugates. Subsequently, 1 × 10^−6^ M N-hydroxy-succinimide (NHS) and 1 × 10^−6^ M 1-(3-dimethylaminopropyl)-3-ethylcarbodiimide hydrochloride (EDC) were added in solution. The solution was stirred for 12 h. The final AuNR product was recovered by configuration and dispersed in 10 mM PBS buffer at pH 7.4.

### Nanoparticle characterization

Transmission electron microscopy (TEM) images were taken with a side-entry Philips electron microscope at 120 keV. The AuNRs were diluted to nanomolar concentrations followed by casting onto the copper grids (200 mesh) with standard carbon-coated Formvar films (200–300 Å). The samples were dried in air for the TEM measurements. The distributions of nanoparticle sizes were analyzed with Scion Image Beta Release 2.

Absorption spectra were recorded on a Hewlett Packard 8453 spectrophotometer. Ensemble fluorescence spectra were recorded on a Cary Eclipse Fluorescence Spectrophotometer.

Fluorescence imaging measurements were conducted on a time-resolved scanning confocal microscope (MicroTime 200, PicoQuant) which consists of an inverted confocal microscope coupled to a high-sensitivity detection setup. A single mode pulsed laser diode (470 nm, 100 ps, 40 MHz) was used as the excitation source. An oil immersion objective (Olympus, 100×, 1.3 NA) was used to focus the laser beam on the sample and to collect the emission. The emission passed through a dichroic mirror, focused onto a 75-µm pinhole for a spatial filtering, and recorded on a single photon avalanche diode (SPAD) (SPCM-AQR-14, Perkin-Elmer Inc.). A longpass filter over 750 nm was used to eliminate the residual excitation signals. The data were collected with a TimeHarp 200 board and stored in time-tagged time-resolved mode (TTTR).

### Conjugation of ICG-Au complexes with cells and their cytotoxicity

ICG-Au complexes were conjugated on HeLa cells for fluorescence cell imaging. The HeLa cells were dispersed in Dulbecco’s modified Eagle’s medium (DMEM), supplemented with 10% fetal bovine serum (FBS), and subsequently grown on a 6-well glass coverslip incubated at 37 °C/5% CO_2_/95% humidity for 48 h. The cells were then fixed with 4% paraformaldehyde in 10 mM PBS buffer at pH 7.4 for 30 min at 4 °C. The fixed cells were washed twice with 10 mM PBS buffer followed by incubating with 0.5 nM ICG-Au in 10 mM PBS buffer for 30 min. The samples were rinsed with 10 mM PBS-Mg buffer, dried in air, and stored at 4 °C. The imaging of LNP conjugated-cell samples were performed on the time-resolved confocal microscope.

Cytotoxicity was tested on live HeLa cell using calcein AM assay. Briefly, the HeLa cells were grown in a 6-well glass coverslips for 48 h as described. The cells were washed twice with 10 mM PBS buffer followed by adding 1 μM Calcein AM solution. 0.3 and 3 nM Au nanoparticle solution was added and the cells were continuously cultured in the incubator. The images of live HeLa cells stained with calcein AM were acquired on the confocal microscope at different time intervals with a bandpass filter of 514/30 nm using a 443 nm laser diode as excitation source. The images of dead cells were identified by their stronger autofluorescence on other channel with a longpass filter of 655/20 nm using a 640 nm laser diode as excitation source. The cell images were counted at single cell level and analyzed for the cell viability.

### Small animal tomography measurements

The ICG-Au complexes were tested as imaging contrast agents for fluorescence small animal imaging. Typically, 5–6 weeks nude mice were first euthanized under deep anesthesia. Removing the hair on the belly, the mice was injected by 0.1 mL of 10 mM PBS buffer solution containing 0.5 nM ICG-Au complexes below the surface of the mice skin. Subsequently, the same volumes of ICG- HSA conjugate (concentration = 3 nM) in 10 mM PBS buffer solution and blank in 10 mM PBS buffer solution were also respectively injected at different sites of same mice. Fluorescence small animal imaging were performed on a Xenogen IVIS-200 system with a bandpass filter of 665–695 nm for the background, a bandpass filter of 710–760 on the excitation side, and a bandpass filter of 810–875 nm on the emission side. A 150 W laser at 750 nm was used as the excitation source. Total photon flux (photons/s) over the measurement was calculated and corrected for tissue depth by the spectral imaging using Living Image 3.0 software (Xenogen). Small animal imaging measurements were conducted under approved IRB protocol from University of Maryland School of Medicine.

## Abbreviations

NFF: near-field fluorescence
NIR: near-infrared
ICG: indocyanine green
AuNR: gold nanorod
LNP: luminescent nanoparticle
CTAB: cetyltrimethylammonium bromide
HSA: human serum albumin
PEG: polyethylene glycol
TEM: transmission electron microscope
EDC: 1-(3-dimethylaminopropyl)-3-ethylcarbodiimide hydrochloride
